# Unusual Presentation of a Case of Upper Limb Lymphedema

**DOI:** 10.1097/GOX.0000000000002137

**Published:** 2019-04-01

**Authors:** Hazim Hussein, Hisham Burezq

**Affiliations:** From the * Babtain Centre of Burn and Reconstructive Surgery, Kuwait; † Al Babtain Centre for Burn, Plastic and Reconstructive Surgery, Ibn Sina Hospital, Sabah Health Area, Kuwait.

## Sir,

I would like to introduce an unusual presentation of a case of upper limb lymphedema post axillary clearance after mastectomy for breast cancer. A 52-year-old woman presented to us with a huge mass in the right arm, which started 4 years post axillary clearance. The mass increased gradually in size. It was well defined, heavy, nonpulsatile, noncompressible, and firm with peau d’orange appearance of the skin originating from the lower pole of the right arm. Her main complaint was heaviness with shoulder and neck pain. Both hand and forearm were moderately edematous. Lymphoscintigraphy revealed complete absence of lymphatic channels in the arm. The mass was resected successfully, and the defect was covered adequately by mobilizing local skin flap. Only 28% of cases of postaxillary clearance will be presented by upper limb lymphedema^1^ because there is a communication between superficial and deep lymphatics in the side of axillary clearance that may facilitate lymph drainage in these situations, illustrating why some cases are not presenting with lymphedema postoperative.^2^

**Fig. 1.**
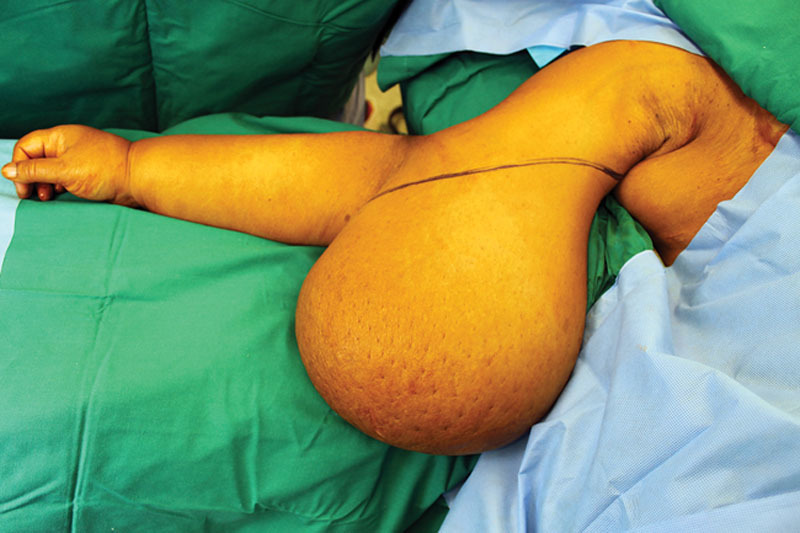
Preoperative photograph.

**Fig. 2.**
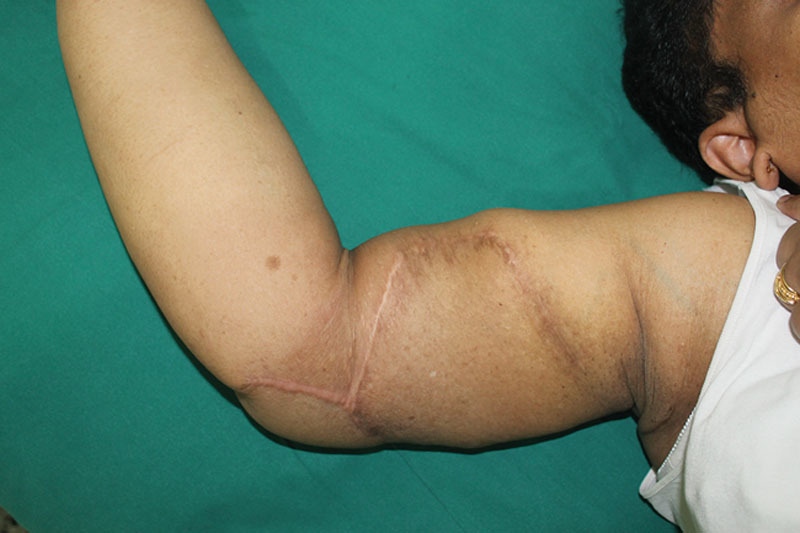
Six months postoperative photograph.

In our case, there is an unusual presentation of lymphedema in terms of isolated huge swelling in the arm which can be explained by the absence of lymphatic system in the arm which was mentioned before.
